# Sensing Properties of Pd-Loaded Co_3_O_4_ Film for a ppb-Level NO Gas Sensor

**DOI:** 10.3390/s150408109

**Published:** 2015-04-07

**Authors:** Takafumi Akamatsu, Toshio Itoh, Noriya Izu, Woosuck Shin, Kazuo Sato

**Affiliations:** 1 National Institute of Advanced Industrial Science and Technology (AIST), Inorganic Functional Materials Research Institute, 2266-98, Anagahora, Shimo-Shidami, Moriyama-ku, Nagoya-shi 463-8560, Japan; E-Mails: itoh-toshio@aist.go.jp (T.I.); n-izu@aist.go.jp (N.I.); w.shin@aist.go.jp (W.S.); 2 Department of Mechanical Engineering, Aichi Institute of Technology, 1247, Yachigusa, Yakusa-cho, Toyota-shi 470-0392, Japan; E-Mail: sato@aitech.ac.jp

**Keywords:** cobalt oxide, gas sensor, nitric oxide, palladium

## Abstract

We prepared 0.1 wt%–30 wt% Pd-loaded Co_3_O_4_ by a colloidal mixing method and investigated the sensing properties of a Pd-loaded Co_3_O_4_ sensor element, such as the sensor response, 90% response time, 90% recovery time, and signal-to-noise (*S/N*) ratio, toward low nitric oxide (NO) gas levels in the range from 50 to 200 parts per billion. The structural properties of the Pd-loaded Co_3_O_4_ powder were investigated using X-ray diffraction analysis and transmission electron microscopy. Pd in the powder existed as PdO. The sensor elements with 0.1 wt%–10 wt% Pd content have higher sensor properties than those without any Pd content. The response of the sensor element with a 30 wt% Pd content decreased markedly because of the aggregation and poor dispersibility of the PdO particles. High sensor response and *S/N* ratio toward the NO gas were achieved when a sensor element with 10 wt% Pd content was used.

## 1. Introduction

Human breath contains many main gases such as N_2_, CO_2_, O_2_ and minor gases such as inflammable gases (CO, CH_4_, and H_2_), and volatile organic compounds (VOCs) [1,2,3,4,5]. Some gases in the human breath are considered as biomarkers for diseases. For example, the breath of lung cancer patients includes aldehydes such as nonanal [3], and nitric oxide (NO) in human breath increases in airway inflammatory disorders such as asthma in adults and children [4,5]. Some biomarker gases in human breath are present at very low concentrations in the range from several parts per billion (ppb) to parts per million (ppm). Thus, monitoring human breath is one of the best non-invasive screening tests for early diagnosis, but this process requires analysis equipment with sufficient accuracy at the ppb level.

NO in human breath has been measured according to the American Thoracic Society recommendations [6] using a chemiluminescence analyzer (model 280i NO analyzer; Sievers, Boulder, CO, USA) and is expressed in ppb. Because the analyzer is bulky and needs an ozone supply, effective, inexpensive, and miniaturized systems for the detection and quantification of NO are required [7]. Gas sensors have been considered promising candidates for NO measurement in human breath owing to their low cost, compact size, and direct electronic interface [8,9]. Many types of NO gas sensors consisting of semiconductors have been investigated [8,9,10,11,12,13,14,15]. Among the metal–oxide–semiconductors, the n-type semiconductors, specifically those based on highly sensitive WO_3_, are promising candidates that can be used to detect NO gas. We have reported the NO and NO_2_ sensing (0.5–5 ppm in air) properties of a p-type Co_3_O_4_ gas sensor [13]. The Co_3_O_4_ gas sensor showed low sensor resistance of below 1 kΩ and the NO sensor responses were higher than the NO_2_ sensor responses. This result suggested that the peripheral circuit used to measure the sensor resistance and the device cost can be reduced. Although the sensor responses of a Co_3_O_4_-based gas sensor at 0.5–5 ppm NO have been investigated, the sensor responses to several hundred ppb NO have not been studied. Moreover, the Co_3_O_4_-based gas sensor showed a low sensor response, even at 0.5 ppm NO and could not accurately detect NO concentrations in several hundred ppb. The addition of metal or metal–oxides in the metal–oxide–semiconductors is critical in improving the sensor response and response time of metal–oxide–semiconductor-based gas sensors [12,14]. Penza *et al.* reported that the Pd-doped WO_3_-based sensor showed higher sensor response to 440 ppm NO gas than the undoped, Pt-doped, and Au-doped WO_3_-based sensors [12]. In addition, the NO gas sensor must show high NO selectivity against other gases such as H_2_, CO, and CH_4_ in human breath. The H_2_ concentration in human breath has been measured at several tens of ppm level and is higher than that of other gases such as inflammable gases and VOCs [16].

In the present study, Pd-loaded Co_3_O_4_ was prepared by a colloidal mixing method [17] to improve the gas-sensing properties, and the sensor response, response time, recovery time, and signal-to-noise (*S/N*) ratio toward low NO concentration were investigated. To study the possibility of breath analysis using a 10 wt% Pd-loaded Co_3_O_4_ sensor, the sensor responses to 25 and 100 ppm H_2_ in air were investigated as an initial evaluation.

## 2. Experimental

Co_3_O_4_ powder with an average particle size of 20–30 nm was used as a NO gas-sensing material. The Co_3_O_4_ powder was mixed and stirred in ethanol solution. Then, Pd colloid suspension (particle size: 4 nm) was added to the Co_3_O_4_-mixed ethanol solution, and the mixed solution was stirred and dried at 50 °C. The Pd contents were 0 wt%, 0.1 wt%, 1 wt%, 5 wt%, 10 wt%, and 30 wt% relative to the Co_3_O_4_ content. The resulting powders were denoted as Co_3_O_4_, 0.1%Pd/Co_3_O_4_, 1%Pd/Co_3_O_4_, 5%Pd/Co_3_O_4_, 10%Pd/Co_3_O_4_, and 30%Pd/Co_3_O_4_, respectively. The powders were mixed with an organic dispersant, which consisted of a mixture of 10 wt% ethyl cellulose and 90 wt% terpineol, to obtain a paste suitable for air dispensing. The weight ratio of the powder to the organic dispersant was 1:16. The paste was dispensed on a surface-oxidized Si substrate, which consisted of 2.5 × 4 mm^2^ platinum interdigital electrodes with line and space definitions of 15 μm each, using an air-dispenser (FAD320s, Musashi Engineering, Tokyo, Japan) [13,17]. The substrate was baked at 400 °C for 2 h under air to obtain the sensor elements. The Co_3_O_4_ film thickness could be controlled to 2–4 μm by air-dispenser.

The obtained sample powders were characterized by X-ray diffraction (XRD) analysis and transmission electron microscopy (TEM). The XRD analysis was carried out using a RINT 2100V/PC instrument (Rigaku Corporation, Tokyo, Japan) equipped with a copper source (CuKα). TEM was performed using a JEM-2010 instrument (JEOL Ltd., Tokyo, Japan) equipped with an energy dispersive X-ray spectroscope (EDX).

To investigate the NO gas-sensing properties, the sensor element was placed in a test chamber heated to 100–300 °C in an electrical tube furnace. Air was introduced into the chamber for 15 min, and a gas mixture of NO in air was then injected for 15 min. Subsequently, the gas mixture flow was halted and replaced by air injected at a flow rate of 200 mL/min. The NO concentration was controlled to the values of 50, 100, 150, and 200 ppb in air. The resistance among the electrodes of the element in various gaseous atmospheres was measured by two-probe method at 10-s intervals using a K2700 digital multimeter (Keithley). We defined the sensor response value (*S*) using the following equation: *S* = *R_g_*/*R_a_*, where *R_g_* denotes the resistance after the NO gas exposure for 15 min and *R_a_* denotes the average resistance (*μ*) in air for 3 min before the NO gas exposure. The coefficient of variation (*CV*) of *R_a_* was defined as *CV*(%) = μ/σ × 100, where *σ* denotes the *R_a_* standard deviation in air for 3 min. The *S/N* ratio was defined as *S/N* = (*R_g_* − μ)/σ. The H_2_ gas-sensing properties were investigated using the same NO gas-sensing measurement apparatus. The H_2_ gas concentration was controlled to 0, 25, and 100 ppm in air.

## 3. Results and Discussion

**Figure 1:**
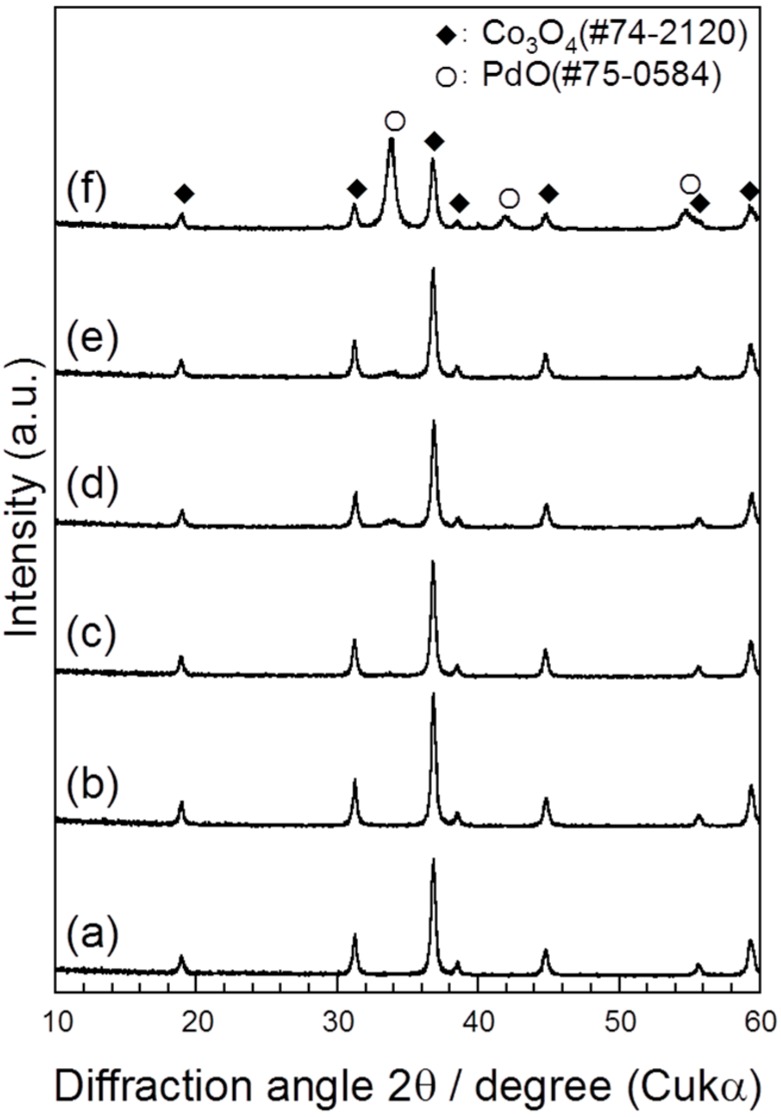
XRD patterns of (**a**) Co_3_O_4_; (**b**) 0.1%Pd/Co_3_O_4_; (**c**) 1%Pd/Co_3_O_4_; (**d**) 5%Pd/Co_3_O_4_; (**e**) 10%Pd/Co_3_O_4_; and (**f**) 30%Pd/Co_3_O_4_ powder after baking at 400 °C for 2 h.

Figure 1 shows the XRD patterns of the Co_3_O_4_, 0.1%Pd/Co_3_O_4_, 1%Pd/Co_3_O_4_, 5%Pd/Co_3_O_4_, 10%Pd/Co_3_O_4_, and 30%Pd/Co_3_O_4_ powder after baking at 400 °C for 2 h. Because the peaks of Co_3_O_4_ (JCPDS card No. 74-2120) were observed in all patterns, no structural changes due to the oxidation or decomposition of Co_3_O_4_ were observed after baking at 400 °C for 2 h. The peaks at 34°, 42°, and 55° were assigned to PdO (JCPDS card No. 75-0584). The palladium, which was loaded by a colloidal mixing method process, was oxidized to PdO. A small hill at 34° was observed in the pattern of 1%Pd/Co_3_O_4_, and this became a peak in the patterns of 5%Pd/Co_3_O_4_, 10%Pd/Co_3_O_4_, and 30%Pd/Co_3_O_4_. The PdO peaks at 42° and 55° were observed in the pattern of 30%Pd/Co_3_O_4_. No PdO peaks were observed in the pattern of 0.1%Pd/Co_3_O_4_ owing to its lower Pd amount. The crystallite sizes (*D*) were calculated using the Scherrer equation: *D* = *kλ*/βcosθ, where *k* (= 0.9) is the shape factor, *λ* is the X-ray wavelength, β is the full width at half maximum of the diffraction peak, and θ is the Bragg diffraction angle. The *D* values of the Co_3_O_4_ peaks at 37° and PdO peak at 34° in 30%Pd/Co_3_O_4_ were estimated to be 19.0 and 11.7 nm, respectively.

**Figure 2:**
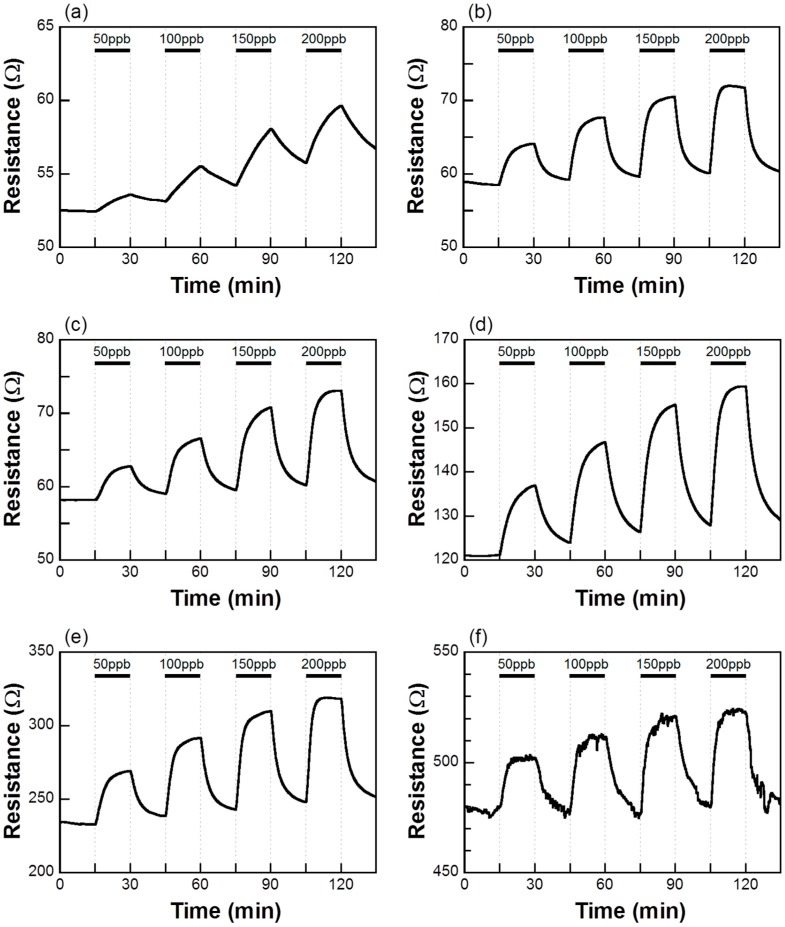
Responses of the sensor element using (**a**) Co_3_O_4_; (**b**) 0.1%Pd/Co_3_O_4_; (**c**) 1%Pd/Co_3_O_4_; (**d**) 5%Pd/Co_3_O_4_; (**e**) 10%Pd/Co_3_O_4_; and (**f**) 30%Pd/Co_3_O_4_ powder during exposure to NO (50, 100, 150, and 200 ppb) in air at 200 °C.

**Figure 3:**
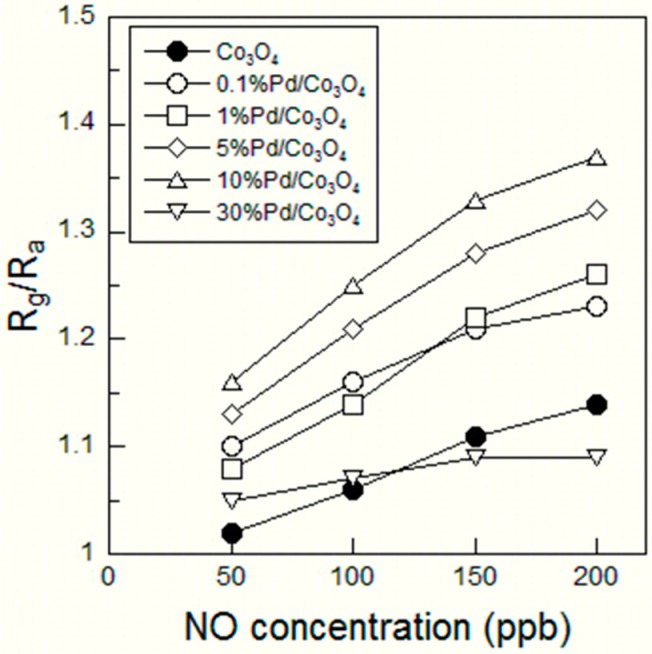
Sensor responses of the sensor element using Co_3_O_4_, 0.1%Pd/Co_3_O_4_, 1%Pd/Co_3_O_4_, 5%Pd/Co_3_O_4_, 10%Pd/Co_3_O_4_, and 30%Pd/Co_3_O_4_ powders as a function of the NO gas concentration.

**Figure 4:**
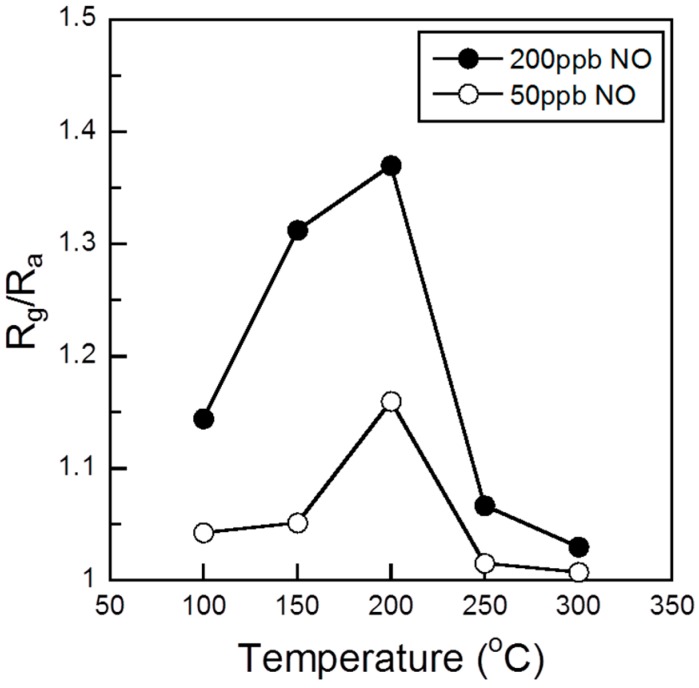
Sensor responses of the sensor element using a 10%Pd/Co_3_O_4_ powder to 50 and 200 ppb NO as a function of the operating temperature.

Figure 2 shows the response of the sensor element using the Co_3_O_4_, 0.1%Pd/Co_3_O_4_, 1%Pd/Co_3_O_4_, 5%Pd/Co_3_O_4_, 10%Pd/Co_3_O_4_, and 30%Pd/Co_3_O_4_ powder during exposure to NO (50, 100, 150, and 200 ppb) in air at 200 °C. The resistance of all sensor elements began to increase when they were exposed to NO gas. In the case of a p-type semiconductor such as Co_3_O_4_ and NiO whose majority carrier is a hole, when the concentration of electrons on the semiconductor surface increases, the resistance of the semiconductor increases because the generated electrons recombine with the holes [18]. When a reductive NO gas is streamed on a p-type semiconductor, the NO gas reacts with the adsorbed oxygen ions on the semiconductor surface, releasing electrons back to the conduction band. Therefore, the resistance of the sensor elements increases toward the NO gas exposure. All sensor elements exhibited a distinct response to NO gas even at 50 ppb. The resistance of all sensor elements increased with the NO gas concentration. The resistance of the NO-exposed Co_3_O_4_ film did not reach *R_a_* even after 15 min of air exposure (see Figure 2a). This result is considered to imply that the adsorbed NO on Co_3_O_4_ film surface did not desorb from the surface during air exposure for 15 min. On the other hand, the resistances of the NO-exposed Pd-loaded Co_3_O_4_ films reached *R_a_* even after 15 min of air exposure (see Figure 2b to Figure 2f). The background resistance signal in Figure 2f was slightly noisy, which might be due to the 30 wt% Pd content. The sensor responses *S*(= *R_g_*/*R_a_*) were plotted with respect to the NO concentration of 50–200 ppb (see Figure 3). All sensor responses linearly increased with the NO gas concentration. The sensor responses increased with the Pd content up to 10 wt% but became low in the 30 wt% Pd content. Since the XRD pattern of the 30%Pd/Co_3_O_4_ showed large PdO crystallite size, the large PdO interfered with the adsorption of the NO onto the Co_3_O_4_, resulting in the decrease of the sensor response. The high sensor responses *S* = 1.16 at 50 ppb NO was obtained for the optimum composition 10%Pd/Co_3_O_4_. Figure 4 shows the sensor responses to 50 and 200 ppb NO of the sensor element using the 10%Pd/Co_3_O_4_ powder as a function of the operating temperature in the range from 100 to 300 °C. The NO sensor response of the 10%Pd/Co_3_O_4_ sensor was the highest at 200 °C.

Table 1 shows the sensing properties of the Co_3_O_4_ and Pd/Co_3_O_4_ sensor elements upon exposure to 50 ppb NO in air at 200 °C. The 90% response time, defined as the time needed to reach 90% of the final steady value of *R_g_* toward 50 ppb NO exposure, and the 90% recovery time, defined as the time needed to reach 90% of *R_a_*, for the Co_3_O_4_ sensor element, were 12.7 and 13.5 min, respectively. On the other hand, the 90% response time and 90% recovery time for the Pd/Co_3_O_4_ sensor elements were approximately 5 min shorter than those for the Co_3_O_4_ sensor element. This result suggests that the loaded PdO provided some kinds of surface modification effects with Co_3_O_4_. Thus, the average resistance μ increased with the Pd content in the films due to this modification effects. Although μ increased due to the Pd loading, all sensor resistances, which were less than 600 Ω, were lower than the resistances of the sensor using WO_3_ and SnO_2_ in our earlier works [13,17].

**Table 1:** Sensing properties of the Co_3_O_4_ and Pd/Co_3_O_4_ sensor elements upon exposure to 50 ppb NO in air. The operating temperature was 200 °C, and the gas flow rate was 200 mL/min.

Sample	90% Response Time (min)	90% Recovery Time (min)	*R_a_*			*R_g_* (Ω)	*S* (*R_g_*/*R_a_*)	*S*/*N* Ratio
			μ (Ω)	σ (Ω)	*CV* (%)			
Co_3_O_4_	12.7	13.5	52.4	0.005	0.010	53.6	1.02	224
0.1%Pd/Co_3_O_4_	8.5	8.6	58.5	0.018	0.031	64.1	1.10	307
1%Pd/Co_3_O_4_	9.3	9.8	58.2	0.007	0.011	62.8	1.08	695
5%Pd/Co_3_O_4_	9.8	10.3	121.1	0.057	0.047	137.0	1.13	278
10%Pd/Co_3_O_4_	7.6	8.0	233.0	0.041	0.017	269.3	1.16	891
30%Pd/Co_3_O_4_	4.7	7.7	478.5	1.147	0.240	502.2	1.05	21

The *R_a_* standard deviation σ and the *CV* value increased with the Pd content. The *S/N* ratio of the Co_3_O_4_ toward 50 ppb NO exposure was 64 and was lower than that of the Pd/Co_3_O_4_ with 0.1 wt%–10 wt% Pd content. The *S/N* ratio of the 30%Pd/Co_3_O_4_ was lower than that of the Co_3_O_4_ because of the noisy background resistance signal. The *S/N* ratio of the 5%Pd/Co_3_O_4_ was lower than that of the 1%Pd/Co_3_O_4_ or 10%Pd/Co_3_O_4_ owing to large *CV* value. The origin of the discontinuity of the *S/N* ratio is unclear at this stage. Further work is necessary to clarify the origin. Following the conventional definition in Kaiser’s work [19], the limit of detection value was defined as the NO concentration at which a sensor response corresponds to *S/N* = 3. Thus, the sensor element with a large *S/N* ratio can detect a low concentration of NO with high reliability. The limit of detection value (*S*/*N* = 3) of the 10%Pd/Co_3_O_4_ sensor was estimated to be 0.66 ppb considering *S*/*N* = 227 at 50 ppm NO, which was lower than that of the 12 ppb value reported in the literature [14]. The suitable Pd content of the sensing material Pd/Co_3_O_4_ was considered to be 10 wt% from the sensor response and *S/N* ratio.

**Figure 5:**
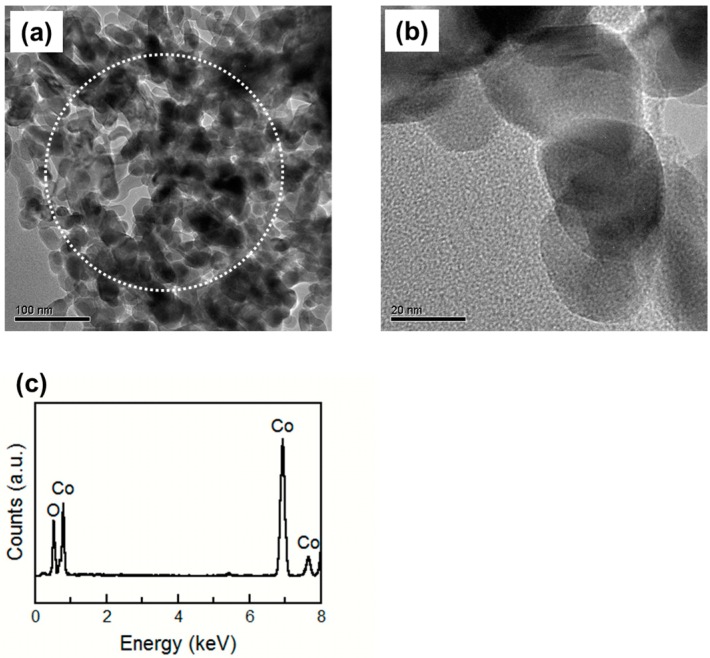
(**a**,**b**) TEM images of the Co_3_O_4_ powder; (**c**) EDX analysis result of the area inside the circle in the (**a**) image. The powder is baked at 400 °C for 2 h.

To examine the reason why the sensor using the 10%Pd/Co_3_O_4_ powder had good sensor properties, TEM observations of the Co_3_O_4_, 10%Pd/Co_3_O_4_, and 30%Pd/Co_3_O_4_ powders were carried out. Figure 5 shows the TEM images and EDX spectrum of the Co_3_O_4_ powder after baking at 400 °C for 2 h. Pale phases were observed, and the grain size of the Co_3_O_4_ powder was approximately 20 nm. EDX spot analysis was carried out at the area inside the circle in Figure 5a. The EDX spectrum showed that the Co_3_O_4_ powder has no impurities such as noble metals.

**Figure 6:**
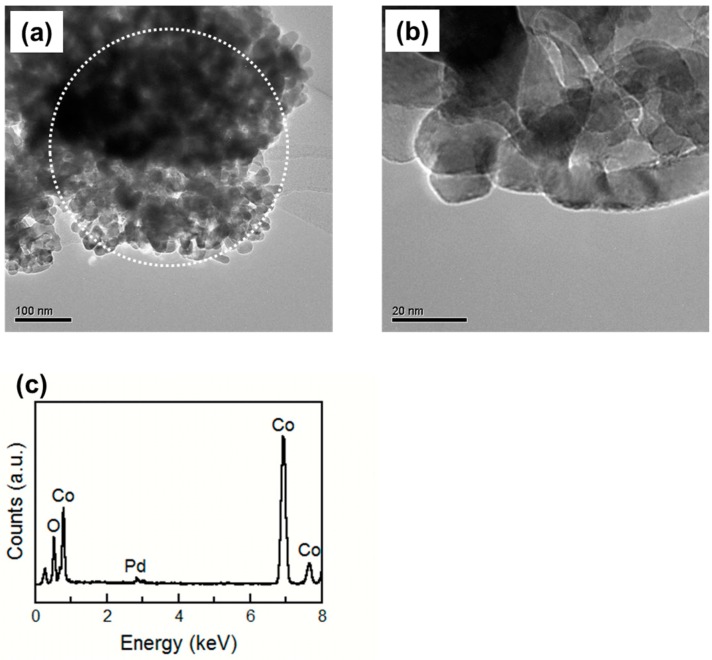
(**a**,**b**) TEM images of the 10%Pd/Co_3_O_4_ powder; (**c**) EDX analysis result of the area inside the circle in the (a) image. The powder is baked at 400 °C for 2 h.

Figure 6 shows the TEM images and EDX spectrum of the 10%Pd/Co_3_O_4_ powder after baking at 400 °C for 2 h. Although no difference was observed in the TEM images between the Co_3_O_4_ and 10%Pd/Co_3_O_4_ powder, Pd peak was confirmed in the EDX spectrum of the 10%Pd/Co_3_O_4_ powder. Matsushima *et al.* reported 0.3–5 wt% Pd-loaded SnO_2_ powder baked at 300 °C in air, and the Pd particles on the SnO_2_ surface became less visible in the TEM image [20], which was considered to be due to the existence of Pd on the SnO_2_ surface as PdO with a lower contrast to SnO_2_. Pd in the 10%Pd/Co_3_O_4_ powder existed as PdO and was not distinguishable from the TEM image.

**Figure 7:**
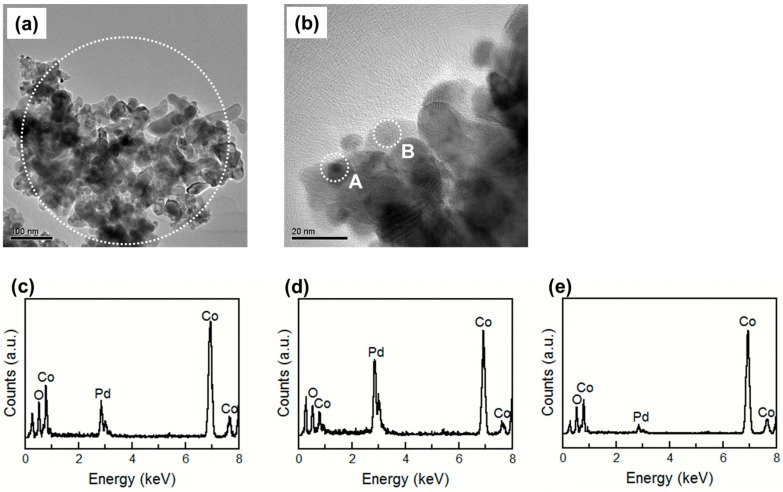
(**a**,**b**) TEM images of the 30%Pd/Co_3_O_4_ powder; (**c**) EDX analysis result of the area inside the circle in the (**a**) image; (**d**) EDX analysis result of the area inside circle A; (**e**) EDX analysis result of the area inside circle B. The powder is baked at 400 °C for 2 h.

Figure 7 shows the TEM images and EDX spectra of the 30%Pd/Co_3_O_4_ powder after baking at 400 °C for 2 h. Figure 7a shows that although no difference was observed among the TEM images with low magnification of the Co_3_O_4_, 10%Pd/Co_3_O_4_, and 30%Pd/Co_3_O_4_ powder, the intensity of the Pd peak in the EDX spectrum increased with the Pd content. Figure 7b shows small dark phases with 5-nm diameter, such as the area inside circle A, and pale phases, such as the area inside circle B. Figure 7d,e shows the EDX spectra of the area inside circles A and B in Figure 7b, respectively. The intensity of the Pd peak in Figure 7c was much lower than that in Figure 7d and higher than that in Figure 7e. This result showed that the aggregation of the PdO particles was formed at the area inside circle A, and the 30%Pd/Co_3_O_4_ powder had poor dispersibility of the PdO particles. Because of the aggregation and poor dispersibility of the PdO particles, the sensor element using 30%Pd/Co_3_O_4_ was considered to show a lower sensor response and *S/N* ratio than the others. No difference was shown between the Co_3_O_4_ crystallite size determined from the Scherrer equation and Co_3_O_4_ particle size obtained by TEM image. However, the size of the PdO aggregation in Figure 7b was smaller than the PdO crystallite size determined from XRD. Because the PdO in the 30%Pd/Co_3_O_4_ powder had a lower contrast to Co_3_O_4_, the PdO aggregation size obtained from the TEM image was smaller than that determined by XRD.

**Figure 8:**
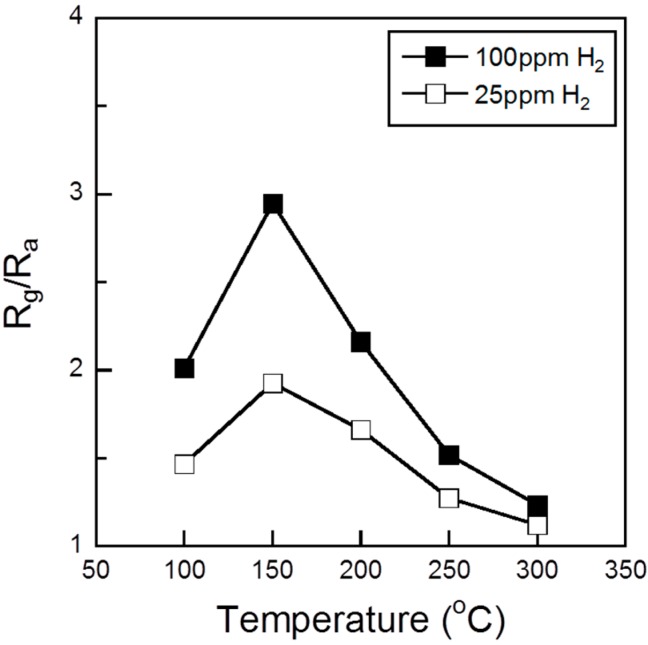
Sensing responses of the sensor element using 10%Pd/Co_3_O_4_ powder to 25 and 100 ppm H_2_ as a function of the operating temperature.

Figure 8 shows the sensing responses of the sensor element using 10%Pd/Co_3_O_4_ powder toward 25 and 100 ppm H_2_ as a function of the operating temperature. When the 10%Pd/Co_3_O_4_ sensor was exposed to H_2_ gas, which is a typical reducing gas, the resistance of the sensor increased. The high sensor responses (*S* = 1.9 at 25 ppm H_2_ and *S* = 3.0 at 100 ppm H_2_) were obtained at 150 °C. This result showed that the 10%Pd/Co_3_O_4_ sensor is not immune to the interference of human breath level H_2_ gas. In addition, the sensor can possibly respond to other inflammable gases and VOCs because semiconductor sensors are sensitive. Therefore, the Pd-loaded Co_3_O_4_ sensor needs to have a gas separation membrane and a filter to remove interference such as H_2_, CH_4_, CO, and H_2_O.

The Pd/Co_3_O_4_ sensor elements showed a good response to 50 ppb of NO in air. Further investigation for improvement of the sensor response is required to detect the sub-ppb levels of NO with sufficient accuracy. To enhance the sensor response, sensor properties using Co_3_O_4_ powder with noble metals such as Pt, Au, and Ag, will be investigated in the future.

## 4. Conclusions

In this paper, we have investigated the gas sensing properties of a sensor element using Pd-loaded Co_3_O_4_ prepared by a colloidal mixing method. The Pd particles in the Co_3_O_4_-based sensing materials existed as PdO particles. The sensor elements with 0.1 wt%–10 wt% Pd content showed shorter 90% response time and 90% recovery time and higher *S/N* ratio than those with no Pd content. However, the sensor response and *S/N* ratio of the sensor element with a 30 wt% Pd content markedly decreased because of the aggregation and poor dispersibility of the PdO particles. The sensor element with 10 wt% Pd content showed a high sensor response (*S* = 1.16) at 50 ppb NO.
